# Goethe as an Anatomist

**Published:** 1899-11

**Authors:** 


### Goethe as an Anatomist.

Galen noted an intermaxillary bone in animals, and also ascribed them to man, but centuries later Camper claimed that the lack of these bones is an essential mark of distinction between man and animals, especially the monkey. Goethe revived the discussion, and, after considerable research, compelled the recognition of the site of the incisors as rudimentary intermaxillary bones. He also tried to prove that the skull is but a higher developed vertebra, and urged that the three portions of the temporal bone should be considered distinct.—*The Journal.*

